# Characterization of migraineurs presenting interictal widespread pressure hyperalgesia identified using a tender point count: a cross-sectional study

**DOI:** 10.1186/s10194-017-0824-0

**Published:** 2017-12-28

**Authors:** Toshihide Toriyama, Tetsuyoshi Horiuchi, Kazuhiro Hongo

**Affiliations:** 1 Toriyama Clinic, Hachiman 187-1, Komoro, Nagano, Japan; 2 Department of Neurosurgery, Shinshu University School of Medicine, Asahi 3-1-1, Matsumoto, Nagano, 390-8621 Japan

**Keywords:** Migraine, Tender point, Hyperalgesia, Allodynia, Fibromyalgia, Pain thresholds, Central sensitization

## Abstract

**Background:**

Migraineurs exhibit pain hypersensitivity throughout the body during and between migraine headaches. Migraine is classified as a central sensitivity syndrome, typified by fibromyalgia showing widespread pressure hyperalgesia determined by a tender point. This study was performed to examine whether: 1) there is a subgroup of episodic migraineurs with widespread pressure hyperalgesia during and between attacks; 2) if such a subgroup exists, what is the prevalence and what is the difference between groups with interictal widespread hyperalgesia and acute allodynia regarding the demographic and clinical characteristics of migraine.

**Methods:**

This was a cross-sectional study. A total of 176 consecutive episodic migraineurs and 132 age- and sex-matched controls were recruited. The presence of widespread pressure hyperalgesia was investigated using manual tender point survey. To classify a subject’s response as widespread pressure hyperalgesia, the cutoff value for responders was defined as the positive tender point count below which 95% of controls responded.

**Results:**

Based on the number of positive tender points in controls, the cutoff value of tender point count for pressure hyperalgesia responders was 7. Of the 176 subjects, interictal widespread pressure hyperalgesia and acute allodynia were observed in 74 (42%) and 115 (65.3%) patients, respectively. Univariate analysis indicated that risk factors associated with interictal widespread pressure hyperalgesia were female gender, younger age at migraine onset, higher frequency of migraine attacks, severe headache impact, cutaneous allodynia and depression. Multivariate logistic regression analysis confirmed that independent risk factors associated with interictal widespread pressure hyperalgesia were female gender, higher frequency of migraine attack and younger age at onset.

**Conclusion:**

Interictal widespread pressure hyperalgesia was common (42%) in the episodic migraineurs and was associated with younger age at onset, female gender, and higher frequency of headache, but not duration of migraine illness. Presence of interictal widespread pressure hyperalgesia is assumed to be an indicator of genetic susceptibility to migraine attacks. We expect that a tender point count, as an alternative to quantitative sensory testing, will become useful as a diagnostic indicator of interictal hyperalgesia in migraineurs to predict susceptibility to migraine attacks and to permit tailored treatment.

**Electronic supplementary material:**

The online version of this article (10.1186/s10194-017-0824-0) contains supplementary material, which is available to authorized users.

## Background

Burstein et al. [1] first reported increased pain sensitivity during migraine attacks, with migraine patients showing pain resulting from innocuous and nocuous stimuli to the normal skin or the scalp during and between attacks. This pain is referred to cutaneous allodynia (CA) [2–5] and hyperalgesia [6, 7]. CA is a common symptom in migraine, with an estimated prevalence of around 60% [2–5] and has been proposed as a clinical manifestation of central sensitization [1, 8–10]. Early studies on allodynia were conducted using quantitative sensory testing (QST). Recently, a structural questionnaire [2, 11] has been developed and widely implemented as a substitute for QST to diagnose CA.

Lowering of the pain threshold leads to experimental pain (hyperalgesia) in migraineurs compared to healthy controls [6, 7, 12–19]. While a few studies indicated hyperalgesia to experimental pain during migraine attacks [1, 11], most studies investigated hyperalgesia between attacks [6, 7, 12–19] to avoid the influence of acute allodynia. Hyperalgesia is also observed in and around the trigeminocervical region [7, 11–17, 19] and throughout the body [6, 18]. During migraine attacks, it is difficult to evaluate hyperalgesia using a questionnaire because hyperalgesia is a strong pain induced by potentially noxious stimuli and is rarely experienced in mundane activity. Therefore, studies on interictal hyperalgesia have mostly been performed in tertiary headache centers using QST.

Although QST is the gold standard method for assessing pain hypersensitivity, it is not suitable for practical use in primary or secondary care clinics. As it requires special equipment, skill, and a lengthy examination time, it is not feasible in large population studies [11]. Therefore, a practical tool that can be used in place of QST to diagnose interictal hyperalgesia and CA is needed. The manual tender point survey (MTPS) represents a method to evaluate pressure hyperalgesia in fibromyalgia (FM) [20, 21]. Palpation of these tender points (TPs) can produce a pressure pain response, allowing examination of local and widespread hyperalgesia. The comorbidity of migraine and FM is well known [10], and migraine is classified as a central sensitivity syndrome [22–26] by typified FM showing widespread pressure hyperalgesia (WPH) determined by tender point count (TPC). A common mechanism seems to underlie the widespread pressure hyperalgesia (WPH) in both FM and migraine patients [10]. Therefore, we considered it reasonable to evaluate WPH in migraine patients using MTPS.

This study was performed to examine whether: 1) a subgroup of episodic migraineurs with WPH exists; 2) if such a subgroup exists, what are the differences in the demographic and clinical characteristics of migraine, including positive TP, between groups with interictal WPH and CA.

## Methods

### Study design and setting

Migraineurs were recruited for the cross-sectional survey from March 2015 to September 2016 at Toriyama Clinic (local primary and secondary headache clinic in Komoro City, Nagano Prefecture, Japan, with a target population of around 100,000). All subjects had a standardized interview and careful clinical examinations were conducted by the first author to determine inclusion and exclusion criteria. This study was approved by the institutional review board of Shinshu University School of Medicine (approval number 3552-1). All patients provided informed consent prior to enrollment in the study.

### Participants

Consecutive patients were considered eligible for inclusion in this study using the following inclusion criteria: episodic migraine as defined by the International Classification of Headache Disorders (ICHD)-III β criteria [27], aged 18-65 years, and subjects that had been migraine-free for at least 48 h to avoid the influence of migraine-related acute allodynia. Exclusion criteria were as follows: comorbid diagnosis of other primary or secondary headache, including trigeminal/occipital neuralgia, sinusitis, chronic migraine or medication overuse headache; history of cervical spondylosis; comorbid diagnosis of any local or systemic disease that may cause headache; and chronic widespread/regional pain or fibromyalgia. All patients were permitted to take migraine abortive and prophylactic drugs.

### Clinical evaluation

The comprehensive interview form included diagnostic questions based on the ICHD)-III-β criteria [27] for diagnosis of migraine, demographic characteristics, and migraine-specific features. Migraine-specific variables included migraine subtype, age at onset, duration of migraine illness, duration of migraine attacks, migraine attack frequency, headache intensity, headache disability, presence of cutaneous allodynia, presence of depression, migraine-associated symptoms, and use of headache medicine. The age at onset was divided into 5 categories. Duration of migraine history was divided into four categories. Duration of headache attack was classified into four classes. We measured migraine frequency by the number of migraine days per year and used four frequency classes. Medication intake was determined as clinical history. For women patients, no information of menstrual cycle was obtained in this study.

### Measurements

As no standardized measures are currently available to determine the presence of interictal WPH without using QST, the existence of WPH using TPC by MTPS for FM was investigated [28]. The author (TT) performed MTPS in a non-blinded manner. MTPS was performed systematically over the 18 FM tender points according to the published guidelines [28] in all migraineurs and controls. Digital pressure was incremented until the examiner’s nail bed became white (force of about 4 kg) [29]. Participant’s pain response at each site was classified as no pain, mild pain (complaint of pain without grimace, flinch, or withdrawal), moderate pain (pain plus grimace or flinch), and severe pain (pain plus marked flinch or withdrawal) [30]. Although TP with mild pain was rated as positive TP in the original guidelines [28], a mild pain response was considered potentially ambiguous because the objective of this study was to evaluate the relationships between WPH and migraine characteristics. Therefore, the cutoff response was set as greater than or equal to moderate pain according to Amris et al. [31] who suggested that a hyperalgesic pain response at palpation should be required for TP to be considered a primary identifier of pain hypersensitivity. Thus, the sum of the positive TPs, above cutoff thresholds set at ≥moderate, was defined as the TPC in this study. The maximum TPC was 18 (9 designated spots × right/left). With regard to the order of examinations, the first author checked the front of the body (in the order low cervical, second rib, lateral epicondyle and knee), and then examined the back of the body (in the order occiput, trapezius, supraspinatus, gluteal and greater trochanter) with the subject in a standing posture. In this study, only one examiner (TT) rated all TPs, indicating negligible inter-rater reliability. Prior to the study, a digital weight scale was used to train the rater.

As there were no standardized measures for evaluating WPH using MTPS, selection of threshold of TPC for WPH was needed. We recruited headache-free healthy controls to define TPC cutoff values for WPH in migraineurs. Control subjects that visited to Toriyama Clinic for health checkups were examined for TPs by the same rater blinded to their pain. After obtaining information on the current pain, subjects complaining of headache and other pain were excluded. To classify a subject’s response as WPH in a standardized manner, the cutoff value for WPH responder was defined as the TPC below which 95% of control subjects responded. Patients were classified into the migraine with interictal WPH subgroup, if they had a positive TPC above the threshold.

### Allodynia symptom checklist

The comprehensive interview included a 12-item questionnaire similar to the items of the validated Allodynia Symptom Checklist [2]. Migraine patients that answered “yes” on two CA items were scored as allodynic [3].

### Numeric rating scale

The intensity of headache was derived based on a numerical rating scale [32], with 0 representing “no pain” and 10 representing “the worst pain possible.” Mild headache was defined by scores 1–4; moderate by scores 5–7; and severe by scores 8–10 [33].

### Headache impact test

Headache Impact Test-6 [33, 34] was used to measure headache-related disability. Four groups have been derived to aid in the interpretation of Headache Impact Test −6 scores. In this study, patients were divided into two groups according to headache-related disability: “no severe impact” (score < 60) and “severe impact” (score ≥ 60).

### Self-rating depression scale

Depressive symptoms were evaluated by the self-rating depression scale [35]. Patients were diagnosed as having depression, if they had a self-rating depression scale score ≥ 48 [36].

### Statistical analysis

Descriptive data are expressed as means ± standard deviation or percentages. Differences in proportions were tested using the x^2^ tests or Fisher’s exact test as appropriate. Differences in continuous data between the groups were analyzed by using Student’s t-test for normally distributed variables. The Mann-Whitney U test was used to compare differences between non-normally distributed variables. Logistic regression analysis was used to test which variables were independently associated with icterictal WPH. In logistic regression analysis, all variables for which the *P* value was less than 0.15 on the univariate analysis were included in the model, and variables with lower significance (higher P value) were sequentially removed from the model. To confirm whether the MTPS was administered consistently throughout the study period, the Mann-Whitney U-test was used to compare differences between TPC of the first half of the patients and those of the last half of the patients. Spearman’s correlation coefficient was used to investigate the test-retest reproducibility of MTPS in those examined on the initial and second interictal TPC at intervals of more than 1 month to within 6 months. Kappa values was used to investigate the intra-rater agreement of the initial and second diagnoses for positive WPH in the same patients with no change in medication at the same intervals described above. All *P*-values were two-sided, and *P* < 0.05 was taken to indicate statistical significance. All statistical analyses were performed with the free software, EZR [37].

## Results

A total of 202 potentially eligible episodic migraineurs examined between migraine attacks during the study period were recruited. Twenty-six patients were excluded from the study due to medication-overuse headache (*n* = 7), comorbidity of tension-type headache (*n* = 10), occipital neuralgia (*n* = 6), parotitis (n = 1), chronic widespread pain (n = 1) and hypertension (n = 1). Finally, 176 episodic migraineurs (mean age = 41.4 ± 8.0 years, 84.1% women) were enrolled in the study. A total of 132 healthy and headache-free, age- and sex-matched controls (mean age = 40.3 ± 13.0 years, 84.1% women) were also enrolled. The mean TPC of migraineurs (6.3 ± 4.3) was significantly higher than that of healthy controls (1.7 ± 2.4) (*P* < 0.001, Mann-Whitney U test) (Table 1). There were significant differences in the mean TPC between female and male controls and between female and male migraineurs (Table 2).

**Table 1:** Comparison between the positive tender point (TP) count in migraineurs and those of controls

	Migraineurs *n* = 176	Controls *n* = 132	*p* value
Positive TP count	6.3 ± 4.3	1.7 ± 2.4	*p* < 0.001^u^

Values are absolute numbers and mean ± SD

^u^Mann-Whitney U test

**Table 2:** Comparison between the positive TP count in migraineurs and those of controls separated by gender

	Migraineurs *n* = 176		*p* value	Controls *n* = 132		*p* value
Gender	Female (*n* = 148)	Male (*n* = 26)		Female (*n* = 111)	Male (*n* = 21)	
Positive TP count	6.6 ± 4.4	4.4 ± 3.4	0.012^u^	1.8 ± 2.5	0.8 ± 1.9	0.028^u^

**Fig. 1:**
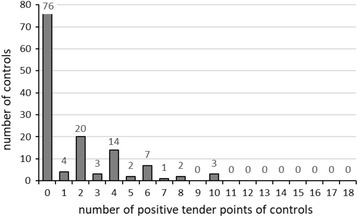
Distribution of number of positive tender point of controls. The cutoff value for WPH responder was defined as the total TPC below which 95% of control subjects responded. This cutoff value was 7

**Fig. 2:**
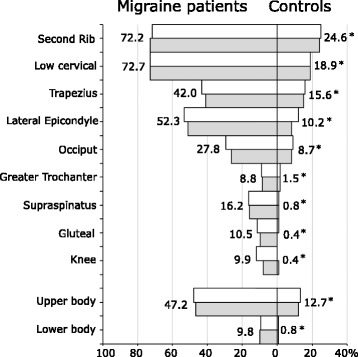
The percentage of migraine patients (left bars) and controls (right bars) with positive tender point sites examined, arranged in sequence from highest to lowest for controls. White bars and gray and bars indicate right and left, respectively. * *p* < 0.001, χ2 test

Figure 1 shows the distribution of the number of positive TPC of controls. The cutoff value for WPH responder was defined as the total TPC below which 95% of control subjects responded. As this cutoff value was 7, patients reporting TPC ≥ 7 were defined as migraineurs with WPH. Figure 2 presents the percentages of migraine patients (left bars) and controls (right bars) with positive TP sites examined, arranged in order from highest to lowest for controls. White and gray bars indicate the right and left sides, respectively. There was no left-right difference in positive TP at paired sites in both migraineurs and controls. (*P* > 0.48, Fisher’s exact test). A significantly high frequency of positive TP on every nine pairs of TPs was found between migraineurs and controls (*P* < 0.001, χ^2^ test). The frequency of positive TP in the upper body was significantly higher than that in the lower body (*P* < 0.001, χ^2^ test) in both controls and migraineurs (Fig. 2, lower). During the study period, the median of TPC in 88 patients was not significantly different between the beginning (6; minimum = 2, maximum = 17) and the end (6; minimum = 4, maximum = 16). The test-retest reproducibility of the TPC was examined in 27 patients. Evaluation of test-retest reproducibility produced a Spearman’s correlation coefficient of 0.86 (P < 0.001), indicating a strong correlation resulting in good test-retest reproducibility. Kappa values were used to investigate the intra-rater agreement of the initial and second diagnoses for positive WPH in 27 patients. Kappa values showed substantial intra-rater agreement in WPH diagnosis (κ values: 0.76~1.07). Table 3 shows the baseline characteristics of 176 migraine patients, separated by the presence of interictal WPH (left) and acute CA (right).

**Table 3:** Baseline characteristics of 176 migraine patient, separated by the presence of interictal widespread hyperalgesia (left) and acute allodynia (right)

	Migraine without IWH	Migraine with IWH	*p* value	Migraine without ACA	Migraine with ACA	*p* value
	*n* = 102	*n* = 74		*n* = 61	*n* = 115	
Variables						
General variables						
Female gender	80 (78.4%)	68 (91.9%)	***0.027*** ^x^	45 (73.8%)	103 (89.6%)	***0.012*** ^x^
Age (years)	39.9 ± 9.7	37.4 ± 10.6	0.106^t^	39.4 ± 11.0	38.5 ± 9.7	0.595^t^
Migraine-specific variables						
Migraine type			0.703^x^			***0.038*** ^x^
Migraine without aura	62 (60.8%)	42 (56.8%)		43 (70.5%)	61 (53.0%)	
Migraine with aura	40 (39.2%)	32 (43.2%)		18 (29.5%)	54 (47.0%)	
Age at migraine onset (years)						
less than 15	18 (17.6%)	22 (29.7%)	***0.043*** ^x^	13 (21.3%)	27 (23.5%)	***0.032*** ^x^
15-19	26 (25.5%)	20 (27.0%)		9 (14.8%)	37 (32.2%)	
20-24	17 (16.7%)	12 (16.2%)		9 (14.8%)	20 (17.4%)	
25-29	7 (6.9%)	9 (12.2%)		8 (13.1%)	8 (7.0%)	
30 or more	34 (33.3%)	11 (14.9%)		22 (36.1%)	23 (20.0%)	
Migraine duration (years)						
less than10	27 (26.5%)	15 (20.3%)	0.351^x^	22 (36.1%)	20 (17.4%)	**0.027** ^x^
10-19	35 (34.3%)	33 (44.6%)		18 (29.5%)	50 (43.5%)	
20-29	27 (26.5%)	14 (18.9%)		15 (24.6%)	26 (22.6%)	
30 or more	13 (12.7%)	12 (16.2%)		6 (9.8%)	19 (16.5%)	
Duration of headache attack (hours)			0.884^x^			0.456^x^
12 or less	46 (45.1%)	35 (47.3%)		30 (49.2%)	51 (44.3%)	
13-24	40 (39.2%)	25 (33.8%)		24 (39.3%)	41 (35.7%)	
25-48	9 (8.8%)	8 (10.8%)		3 (4.9%)	14 (12.2%)	
more than 48	7 (6.9%)	6 (8.1%)		4 (6.6%)	9 (7.8%)	
Frequency of headache (no/year)			***0.005*** ^x^			***0.011*** ^x^
1-6	23 (22.5%)	6 (8.1%)		16 (26.2%)	13 (11.3%)	
7-12	20 (19.6%)	11 (14.9%)		11 (18.0%)	20 (17.4%)	
13-54	51 (50.0%)	40 (54.1%)		31 (50.8%)	60 (52.2%)	
more than 54	8 (7.8%)	17 (23.0%)		3 (4.9%)	22 (19.1%)	
Headache intensity			0.621^f^			0.486^f^
Mild	4 (3.9%)	1 (1.4%)		2 (3.3%)	3 (2.6%)	
Moderate	40 (39.2%)	28 (37.8%)		27 (44.3%)	41 (35.7%)	
Severe	58 (56.9%)	45 (60.8%)		32 (52.5%)	71 (61.7%)	
Headache disability (HIT-6)			0.056^x^			***0.004*** ^x^
severe (60 or more)	56 (54.9%)	52 (70.3%)		28 (5.9%)	80 (69.6%)	
Acute cutaneous allodynia (yes)	59 (57.8%)	56 (75.7%)	***0.022*** ^x^	–	–	
Interictal WPH (yes)	–	–		18 (29.5%)	56 (48.7%)	***0.022*** ^x^
Migraine-associated symptoms						
Nausea/Vomiting	96 (94.1%)	69 (93.2%)	1^f^	58 (95.1%)	107 (93.0%)	0.75 ^f^
Photophobia	76 (74.5%)	62 (83.8%)	0.197^x^	37 (60.7%)	101 (87.8%)	***<0.001*** ^x^
Phonophobia	62 (60.8%)	56 (75.7%)	0.056^x^	27 (44.3%)	91 (79.1%)	***<0.001*** ^x^
Osmophobia	44 (43.1%)	37 (50.0%)	0.454^x^	21 (34.4%)	60 (52.2%)	***0.037*** ^x^
Depression (SDS48 or more)	15 (14.7%)	23 (31.1%)	***0.016*** ^x^	10 (16.4%)	28 (24.3%)	0.304^x^
Use of medication						
Use of acute medication	14 (13.7%)	13 (17.6%)	0.627^x^	9 (14.8%)	18 (15.7%)	1^x^
Use of a triptan	45 (44.1%)	33 (44.6%)	1^x^	22 (36.1%)	56 (48.7%)	0.148^x^
Use of prophylactic drugs	6 (5.9%)	11 (14.9%)	0.083^x^	4 (6.6%)	13 (11.3%)	0.455^x^

Values are absolute numbers with corresponding % or means ±SD

*p*-values depicted in bold italic indicate significant difference (*p* < 0.05)

*IWH* interictal widespread hyperalgesia, *ACA* acute cutaneous allodynia, *HIT-6* headache impact test, *SDS* self-rating depression scale

^x^χ^2^ test

^f^Fisher’s exact test

^t^t-test

Of the 176 episodic migraineurs, interictal WPH and acute CA were observed in 74 (42%) and 115 (65.3%) patients, respectively. Univariate analysis indicated that factors associated with increased interictal WPH prevalence were female gender, younger age at migraine onset, higher frequency of migraine attacks, severe headache impact, acute CA and depression (Table 2, left), and that factors associated with increased acute CA prevalence were female gender, presence of aura, younger age at onset, longer duration of migraine history, higher frequency of migraine attacks, severe headache impact, interictal WPH, photophobia, phonophobia and osmophobia (Table 3, right). Multivariate logistic regression analysis confirmed that the following determinants were significantly associated with interictal WPH: (i) female gender (OR = 3.47, 95% Cl = 1.17-10.3); (ii) lower age at onset (≥30 years vs. <15 years) (OR = 0.24, 95% Cl = 0.09-0.66); and (iii) higher migraine attack frequency (≥54 attacks per year vs. 1-6 attacks per year) (OR = 8.75, 95% Cl = 2.34-32.7) (Table 4).

**Table 4:** Multivariate logistic regression model – factors associated with IWPH among those with episodic migraine

Variable		Odds ratio	95% IC	*P*
Gender (female versus male)		3.47	1.17-10.3	0.025
Age at onset migraine (year)				
	15-19 vs. less than 15	0.56	0.23-1.38	0.3
	20-24 vs. less than 15	0.59	0.22-1.62	0.31
	25-29 vs. less than 15	1.38	0.38-4.98	0.63
	30 or more vs. less than 15	0.242	0.09-0.66	0.005
Migraine frequency (attacks per year)				
	7-12 vs. 1-6	1.58	0.46-5.48	0.47
	13-54 vs. 1-6	2.06	0.72- 5.89	0.18
	more than 54 vs. 1-6	8.75	2.34-32.7	0.001

Independent variables with *P* < 0.15 on univariate analysis were introduced into the model: female gender, current age, age at migraine onset, frequency of headache, headache disability, cutaneous allodynia, phonophobia, depression and use of prophylactic medicine

## Discussions

To our knowledge, this is the first study regarding the differences in demographic characteristics and clinical features among migraine subgroups divided according to the presence or absence of interictal WPH. Research leading to mechanistic descriptions of migraine-related acute CA and interictal WPH may lead to treatments that more effectively reduce sensitization and its clinical consequences.

The main findings of this study can be summarized as follows: 1) During the period between attacks, positive TP in migraine patients was significantly higher than that in controls for all nine pairs of tender points. 2) The incidence rates of interictal WPH and acute CA were 42% and 65.3%, respectively. TPC and interictal WPH diagnosis showed good test-retest reproducibility and substantial intra-rater agreement, respectively. 3) Interictal WPH and acute CA were factors related to the mutual increases in frequency of each other. The risk factors of both interictal WPH and acute CA were female gender, younger age at migraine onset and higher frequency of migraine attacks. Depression was a risk factor of interictal WPH but not CA. In contrast, the risk factors of acute CA were presence of aura, longer migraine history, severe headache impact, photophobia, phonophobia, and osmophobia. 4) Multivariate logistic regression analysis indicated that female gender, higher frequency of migraine attacks, and younger age at onset were independent risk factors of interictal WPH.

### Prevalence and distribution of positive TP in migraines and healthy controls

#### Evaluation of positive TP site as the reduced pressure pain threshold (PPT) site

The sites were sorted in descending order of positive TP in the controls, and the order was almost completely consistent with previous reports [38, 39] arranged in ascending order of PPT of healthy subjects using a dolorimeter (Table 5).

**Table 5:** Comparison between sites sorted in descending order of prevalence of positive TP of our controls and sites arranged in ascending order of PPT of controls in previous studies

Descending order of prevalence of positive TP of our controls *n* = 132	Ascending order of PPT of controls of Marques et al. [38] *n* = 54	Ascending order of PPT of controls of Tastekin et al. [39] *n* = 50
Second Rib	Second Rib	Occiput
Low Cervical	Low Cervical	Low Cervical
Trapezius	Occiput	Second Rib
Lateral Epicondyle	Lateral Epicondyle	Knee
Occiput	Trapezius	Trapezius
Greater Trochanter	Knee	Lateral Epicondyle
Supraspinatus	Supraspinatus	Supraspinatus
Gluteal	Gluteal	Greater Trochanter
Knee	Greater Trochanter	Gluteal

Exceptionally, the frequency of positive TP of the knee site where PPT was ranked in the middle in these previous reports was the lowest in the present study, and that of the occiput site where PPT was ranked in the top three in these previous reports was in the middle in the present study. The reasons for these discrepancies should be investigated in future studies. The four lowest ranking sites including the knee were very small, and therefore an increase in the number of controls may result in a switch of the ranking. Although it is unknown why the occipital ranking in our study was not consistent with the results of the two previous studies mentioned above, especially Tastekin et al. [39], one possible reason may be related to ethnic differences [40] in experimental pain responses.

Taking the above findings together, the positive TP site in this study was assumed to generally correspond to the point of reduced PPT measured by QST [41].

#### Prevalence of positive TP

The positive TP in migraineurs was significantly higher than that in controls, which was consistent with previous studies indicating reduced PPT beyond the trigeminal region in migraineurs [7, 13, 16, 18, 19]. The mean TPC number was significantly higher in females than in males in both control and migraine groups (Table 2), consistent with a previous population study showing higher TPC in female subjects [42]. During the period between attacks, the positive TP of migraine patients was significantly higher than that of controls for all nine pairs of TPs. Therefore, migraineurs showed pressure hyperalgesia throughout the body interictally (Fig. 2). In addition, this result was supported by recent studies investigating pressure/mechanical pain sensitivity in migraineurs reporting hyperalgesia beyond the trigeminal nerve area [7, 13, 16, 19] and even throughout the body [18] in migraineurs as compared to healthy controls. In contrast, no difference was reported between the PPT of interictal migraineurs and that of controls in two studies [14, 15]. This conflict may have been due to small sample sizes, the use of different stimulation methods (digital pressure, algometer, von Frey filament), testing at different body sites, different definitions of “interictal,” and/or different inclusion/exclusion criteria (female only, use of prophylactic medications, inclusion of different headache other than episodic migraine).

#### Correlation between the prevalence of positive TP of migraineurs and PPT of corresponding sites in FM patients

The most and the least sensitive sites of episodic migraine patients were similar to those of controls (Fig. 2). We found that the highly positive TP sites in migraine patients were the second rib, the low cervical and lateral epicondyle, consistent with the lower PPT sites of FM patients [38, 39]. In contrast, the low positive TP sites were the gluteal and greater trochanter. These results were also consistent with the sites of higher PPT [38, 39]. Our findings indicated that TPC is negatively correlated with PPT [41].

#### Factors underlying differences in positive TP on sites

Marques et al. suggested [38] that the differences in PPT for each TP site depend on the proximity to the bone surface or the amounts of soft tissue just beneath them. From this perspective, it is difficult to explain why the frequency of PPT at the greater trochanter site, which is vulnerable to bed sores due to proximity to the bone and small amount of underlying soft tissue, was the lowest after to the knee and gluteal region. It also difficult to explain why the frequency of positive TP in the upper body is significantly higher than that in the lower body. As Weinstein reported that the threshold for pressure on each part in normal individuals is lower in the upper body than the lower body [43], it is reasonable to speculate that this result reflects the original mechanoreceptor sensitivity of TP in normal subjects.

#### Similarity between the sites with high prevalence of positive TP in migraineurs and reduced PPT sites of FM patients

In the migraineurs in the present study, the order of positive TP was almost the same as that in descending order in previous reports [38, 39], and in ascending order of PPT in FM patients (Table 6).

**Table 6:** Comparison between sites sorted in descending order of prevalence of positive TP of our migraineurs and sites arranged in descending order of PPT of FM patients in previous studies

Descending order of prevalence of positive TP of our migraineurs *n* = 132	Ascending order of PPT of FM patients of Marques et al. *n* = 54	Ascending order of PPT of FM patients of Tastekin et al. *n* = 50
Low Cervical	Second Rib	Second Rib
Second Rib	Low Cervical	Low Cervical
Lateral Epicondyle	Lateral Epicondyle	Occiput
Trapezius	Occiput	Lateral Epicondyle
Occiput	Supraspinatus	Knee
Supraspinatus	Knee	Trapezius
Gluteal	Trapezius	Supraspinatus
Knee	Gluteal	Greater Trochanter
Greater Trochanter	Greater Trochanter	Gluteal

Similar distributions of positive TP sites in migraine and reduced pressure pain threshold sites of FM suggested that migraine patients were consistently sensitized throughout the body with common mechanisms and factors facilitating sensitization leading to WPH in interictal migraine and FM [10]. In the present study, the TPC of migraineurs was significantly increased throughout the body compared to controls. This finding suggested that the pain threshold of migraineurs was reduced generally. In addition, the pain modulatory system in the central components, but not peripheral components, may be involved in interictal WPH. Schwedt et al. [17] suggested that inadequate nociceptive inhibition at the level of the spinal dorsal horn would be centrally mediated by brainstem areas, as lowered pain thresholds were found beyond the trigeminal innervated region.

### Prevalence of interictal WPH and acute CA and their correlations

#### Prevalence of interictal WPH

The heterogeneity of pain sensitivity within migraineurs suggested that there may be at least two subtypes of episodic migraineurs: one with dysfunction in the pain modulatory system underlying central sensitization and another with more localized pain sensitivity. To identify the former (i.e., migraineurs with WPH), in a standardized manner, the cutoff value for WPH responder was defined as the total TPC score below which 95% of control subjects responded; this cutoff value was 7. Therefore, patients reporting TPC ≥ 7 with positive TP cutoff set at ≥ moderate pain were defined as migraineurs with WPH. In this study, the prevalence of interictal WPH was 42.0% (Table 3). Therefore, a substantial number of migraineurs belonged to the subgroup with significantly high TPC. Migraineurs are not aware of their hypersensitivity to pressure because the intensity of pressure from outside in everyday activity rarely exceeds 4 kg. Thus, experimental pain studies, rather than questionnaires, were necessary to determine whether migraine patients had evidence of interictal WPH. In migraine patients, interictal WPH may be caused by dysfunction of a pain modulatory system sharing FM based on the prevalence and distribution of positive TP [38, 39].

#### Test-retest reproducibility of TPC and intra-rater agreement of interictal WPH diagnosis.

Although the inter-rater reliability of MTPS using TP with a threshold cutoff set at ≥ mild pain response has been confirmed in several studies [21, 44, 45], the inter-rater reliability in this study using TP with the threshold set at ≥ moderate pain response was not verified. Considering responses to pain as recommended previously [46], however, it is reasonable that the diagnosis of positive TP with threshold set at ≥ moderate pain may be less ambiguous [31]. Thus, diagnosis of positive TP using a threshold set at ≥ moderate pain leads to higher inter-rater reliability than a numeric rating scale only. Further verification of this hypothesis is necessary. Even with the above limitations, TPC and interictal WPH diagnosis showed good test-retest reproducibility and substantial intra-rater agreement, respectively. Consequently, although TPC is an incomplete measure compared to QST, TPC with threshold cutoff set at ≥7 and with positive TP cutoff set at ≥ moderate pain may be a practical diagnostic measure of interictal WPH.

#### Prevalence of acute CA

The prevalence of acute CA in this cohort was 65.3%. The incidence of allodynia ranges from 49.7 to 79% [1–5, 47–53]. Consistent with previous large research studies [2–5, 50, 53], the risk factors of acute CA in the present study were female gender [3, 5, 50, 53], younger age at onset [53], longer migraine history [3, 5, 53], presence of aura [2, 5, 53], higher frequency of attacks [2, 3, 50, 53], severe headache impact (disability) [2, 5, 50], photophobia [2, 5], phonophobia [2, 5], and osmophobia [5]. On the other hand, although depression has frequently been reported to be associated with acute CA [3, 4, 50, 53, 54], no such association was observed in the present study. This discrepancy may have been due to the strict selection of episodic migraine in this study with exclusion of tension type headache comorbidity, chronic headache and medication overuse headache, which are well known to be associated with depression [55]. Therefore, the depressed state in our cohort may have been biased toward a homogenized population, and it was therefore assumed to be difficult to find a significant difference in the prevalence of depression between the subgroup with and without CA. It is also necessary to consider the differences in methods of evaluating depression and the influence of ethnic differences [40].

#### Association between interictal WPH and acute CA

We found that interictal WPH and acute CA were related to increases in frequency of each other. This suggested that both are common clinical symptoms due to the pain modulatory system underlying the basis of sensitization [8, 14, 22, 24, 56]. On the other hand, Schwedt et al. [14] reported that there were no significant correlations between interictal PPT and current CA symptom scores (ASC-12). This difference was likely due to the sample size [14]. We assume that, if a difference is detected, there may be a correlation between interictal PPT and ictal allodynia score. On the other hand, Schwedt et al. [14] reported that interictal migraineurs were significantly more sensitive to thermal stimulation compared to controls. While there were differences in stimulus modalities, our findings and those of Schwedt et al. were consistent in that interictal hyperalgesia was associated with acute CA. Tietjen et al. [53] also reported that CA is associated with an increased prevalence of FM (a central sensitivity syndrome with WPH).

#### Factors related to the frequency of interictal WPH and acute CA

##### Factors related to the frequency of both interictal WPH and acute CA

Other factors related to the high frequencies of both interictal WPH and acute CA were female gender, younger age at migraine onset, and higher frequency of migraine attacks. In experimental pain, women had known to have more pain hypersensitivity than men for most pain modalities [57]. With regard to the frequency of maigraines, our study supported the results of Giamberardino et al. [58] who concluded that the level of hypersensitivity is a function of the number of migraine attacks and confirmed the results of de Tommaso et al. [59] who demonstrated a significant correlation between pain at TP and headache frequency in FM comorbid patients. Giamberardino et al. [58] also reported that effective migraine prophylaxis resulted in a significant improvement in FM symptoms (an increase in pain threshold). Our results were in conflict with several previous reports. Palacios-Ceña et al. [18] reported that the headache intensity, but not frequency, of migraine was negatively associated with widespread pressure pain sensitivity. This may have been because about a half of their subjects had chronic migraine and were permitted to use prophylactic drugs, so their headache frequency was likely less than before the intervention. Schwedt et al. [17] reported that the pain thresholds were correlated, not with frequency, but with time interval until the next migraine attack [60]. This discrepancy may have been because the migraine subgroup with interictal WPH was based on the diagnosis determined by the threshold of TPC. The migraine subgroup with interictal WPH may consist of migraineurs with highly reduced pain thresholds, those with a shorter time interval to the next migraine attack [17]. Thus, it is reasonable to consider that both our and the previous results were consistent because a shorter time to next headache would be associated with a higher frequency of headache attacks.

##### Factors related to the frequency of interictal WPH only

The only risk factor related to the frequency of interictal WPH was depression. Croft et al. [42] also concluded that TPC is related to depression. Intriguingly, there was no significant difference in the prevalence of depression between the subgroups with and without acute CA, possibly because of the homogeneity of depressive state in our cohort. We found a significant difference in the prevalence of depression between the subgroups with and without interictal WPH in the same cohort. This suggests that, while both interictal WPH and acute CA may be clinical manifestations of dysfunction of the central pain modulatory system, interictal WPH may be attributable to regions more favoring depression compared to acute CA. As another factor, it is necessary to consider that MTPS itself is strongly correlated with measurement of distress [61].

##### Factors related to the increase in acute CA only

Factors related to the increase in acute CA, but not interictal WPH, were presence of aura, longer migraine history, severe headache impact, photophobia, phonophobia and osmophobia. This suggests that different pathophysiology is involved in interictal WPH and acute CA. It is noteworthy that all prominent symptoms of migraine (aura, photophobia, phonophobia and osmophobia) were related to an increase in acute CA, but not interictal WPH. This result suggests that independent factors may affect interictal WPH or acute CA. The sensitization of different neuronal populations may induce migraine associated with interictal WPH or acute CA.

### Lesions responsible for acute CA

All prominent symptoms of migraine are known to result from activation of the thalamus and/or cortex [62–68] and they are associated with acute CA but not interictal WPH. Acute CA may be associated with dysfunction of regions involving pain modulatory systems affecting sensitization of the thalamus and/or cerebral cortex. In contrast, interictal WPH may be caused by dysfunction of different regions involving pain modulatory systems related to depression.

### Candidate brain regions of interictal WPH

As interictal WPH is a feature of FM, putative candidates for the central nervous system regions involved in the mechanism underlying interictal WPH are presumed to be as follows.

The somatosensory cortex, with cerebral blood flow positively correlated with headache frequency [69], may be one of the regions related to interictal WPH. Hodkinson et al. [69] reported a significant increase in regional cerebral blood flow in the primary somatosensory cortex of migraineurs compared with healthy controls. They showed that the cerebral blood flow in the primary somatosensory cortex was positively correlated with headache attack frequency. However, it was unrelated to the duration of illness or age of the patients. In our study, the distribution of interictal positive TP corresponded to the distribution of homunculus of the primary somatosensory cortex. The prevalence of interictal WPH determined by positive TPC was related to the frequency of headache attacks, but not to current age or duration of disease. Based on these findings**,** the interictal increase in regional cerebral blood flow of the primary somatosensory cortex may cause interictal WPH. Patients with migraine and interictal WPH may include those with a high expectancy and hypervigilance for pain due to the nature of MTPS [61].

Blood oxygenation level-dependent signals of the posterior thalamus elicited by extracephalic skin stimuli were greater during migraine attacks than the interictal period. This suggests that sensitization of trigeminovascular thalamic neurons induces multimodal allodynia and hyperalgesia throughout the body [67]. However, it is unclear whether interictal blood oxygenation level-dependent signals in response to extracephalic skin stimuli in the trigeminovascular thalamic neurons of migraine patients are greater than those of headache-free controls. Any differences in blood oxygenation level-dependent signals of posterior thalamic neurons in response to extracephalic stimuli between migraineurs and controls may be related to interictal WPH. Further investigations are needed to determine whether blood oxygenation level-dependent signals are different between subjects with and without interictal WPH.

Diffuse noxious inhibitory control [70] failure may also be involved in extensive pain hypersensitivity, as chronic migraine patients frequently report diffuse somatic pain [73], suggesting that the same factors that facilitate the evolution from episodic to chronic migraine may also predispose patients to FM comorbidity.

The brainstem involves nuclei closely related to dysfunction of the descending modulatory system [71]. Studies on periaqueductal gray matter [72], rostral ventromedial medulla [73] and nucleus cuneiformis [74] suggested that disorder of descending pain control plays a key role as a mechanism related to interictal hypersensitivity to pain in migraine patients. Moulton and colleagues reported interictal hypofunction of the nucleus cuneiformis in migraine patients using functional MRI [74]. They suggested that nucleus cuneiformis function could potentially serve as a diagnostic measure in migraine patients, even when not experiencing an attack. Based on the viewpoint that migraineurs with interictal WPH belong to a migraine subtype with dysfunction of pain modulatory systems sharing FM, we hypothesize that the brain region including the dysfunctional pain modulatory system underlying interictal WPH is the medulla. Recent morphological studies showed that migraineurs with more severe symptoms of allodynia and lower heat-pain thresholds show greater volume loss in specific brainstem regions (midbrain, medulla, and cerebellar peduncles), providing further evidence that the brainstem has a modulatory function in the development of central sensitization in migraine [75] and brainstem volume reduction is correlated with TPC in migraine subgroup with FM [76]. Notably, in another report indicating brainstem volume reduction in migraine patients, Bilgiç et al. reported that there was no correlation between brainstem volume and duration of disease [77]. As interictal WPH is associated with younger age at onset rather than duration of disease, structural changes in the brainstem associated with interictal WPH, if present, may be related to the process of migraine susceptibility.

Possible structural changes in the brainstem may be not simply a secondary phenomenon and may be also related to a genetically determined structural alterations in the migraine brain [78]. Schwedt et al. [17] suggested that lower pain thresholds beyond the trigeminal region would likely be mediated by brainstem regions of the descending pain modulatory system, such as the rostral ventromedial medulla, periaqueductal gray matter, and/or nucleus cuneiformis. Consistent with this hypothesis, functional imaging studies of migraineurs have shown that these brainstem regions have atypical pain-induced activation and atypical resting state functional connectivity, and implicated these brainstem regions in central sensitization [72, 74, 79].

Among the above mechanisms, we assume that the medulla is substantially involved as a hub structure related to interictal WPH, because similar brainstem volume reduction and structural alteration [75, 76] were observed in migraine and FM, and it includes the serotonergic system from the rostral ventromedial medulla, which has been implicated in migraine and FM pathophysiology [80]. There have been no previous reports regarding the structural changes in the medulla associated with interictal WPH. Further investigations to examine these association are required.

### Possible therapeutic approach to migraine with interictal WPH

Schwedt et al. [17] hypothesized that the mechanisms underlying these lower pain thresholds could also predispose migraineurs to subsequent migraine attacks based on their observation of the positive correlations between pain thresholds and time to next migraine attack. They concluded that interictal hyperalgesia may be a part of the process of migraine development, and not simply a secondary phenomenon suggesting that the mechanisms underlying interictal hyperalgesia may predispose migraineurs to their next migraine attack and may be a marker of migraine activity and a target for treatment. Giamberardino et al. [58] showed that increased migraine frequency enhances both hyperalgesia and spontaneous FM pain reversed by effective migraine prophylaxis. Longitudinal investigations are necessary to determine whether effective migraine prophylaxis leads to reduce interictal WPH. In 1999, Okifuji et al. [81] concluded that extensive dysregulation of pain modulation is important for a substantial minority of recurrent headache patients, who seem to be quite similar to FM patients, and that differential treatment planning targeting generalized hyperalgesia may be useful for more effectively treating headache patients exhibiting generalized hyperalgesia. For migraines with interictal WPH, a possible subtype of migraineurs sharing FM feature, pharmacological and non-pharmacological [10, 82] effective interventions for FM may be considered. Candidate drugs to improve interictal WPH include current agents, such as pregabalin [83] and duloxetine [84], and/or future agents capable of improving the descending modulation system. Diagnosing interictal hypersensitivity to experimental pain using TPC at the primary clinic where headache patients first visit may prompt general practitioners to start tailored treatment considering central sensitization and underlying dysfunction of the pain modulatory system from an early stage of migraine.

### Clinical significance of TPC as a practical alternative for QST to diagnose interictal WPH in migraine

Although the diagnostic value of MTPS is decreasing in the field of FM [85], TPC is worth revaluating as a practical alternative for QST to diagnose interictal WPH in migraine. We expect general practitioners to be able to identify migraine patients with interictal WPH using TPC without QST, like a McBurney sign for the diagnosis of appendicitis. If the primary care physician can diagnose the presence of interictal WPH, many migraineurs will be able to receive tailored treatment considering dysfunction of the pain moderation system underlying interictal hyperalgesia, from the early stage of migraine. Finally, we endorse the conclusion of de Tommaso et al. [86] that “Improved insight into the interictal dysfunction of temporal information processing in individuals with migraine will pave the way for novel therapeutic targets, and could herald improved migraine management.” The first step toward “improved insight” is for general practitioners examining headache patients to routinely diagnose the presence of WPH in migraineurs using practical methods.

### Strengths and limitations

The strengths of this study include the large cohort size, the well-defined migraine status, detailed information on TP characteristics in comparison with healthy controls, and that the presence of WPH was defined in a standardized manner, i.e., the cutoff value of WPH responders was defined as TPC to which 95% of pain-free healthy controls showed a response. Most importantly, this is the first study to investigate the prevalence of interictal WPH in episodic migraineurs using TPC and demonstrating that the independent factors associated with an increased interictal WPH prevalence were female gender, higher frequency of migraine attacks, and younger age at onset. However, this study had some potential limitations. First, only one rater was involved, resulting in lack of evaluation of inter-rater reliability and limited generalizability. Second, the standardized TP assessment was conducted as part of the clinical examination, and therefore the examiner could not be blinded to headache diagnoses of subjects other than controls. It is possible that the examiner would expect hyperalgesia at TP in migraine patients. Therefore, the results must be confirmed in a blinded manner. Third, we recruited our patients from a secondary headache clinic seeking further medical therapies. As refractory patients visiting a tertiary headache center and mild headache patients improving with non-prescription drugs were excluded, the headache intensity, disability, and depression of our cohort may have been biased toward homogenized moderate state compared to those treated in the general population. This may explain why neither interictal WPH nor acute CA was associated with headache intensity. Therefore, further multicenter studies including individuals from the general population would help to reconfirm the results. Fourth, the cross-sectional nature of this study prevented us from drawing conclusions about causality in the relationship between interictal WPH and high attack frequency. Fifth, we did not consider the time interval to next migraine attack. A previous study suggested that episodic migraineurs have reduced pain thresholds in the 24 h prior to migraine onset [60]. Although we cannot entirely exclude this as a confounder, it is unlikely that a substantial proportion of our subjects (median frequency and mean duration were 2.0 attacks month and 24 h/attack, respectively) developed a migraine within 24 h of examination.

### Generalizability

Although this was a non-blinded study by one rater in a small city in Japan, the demographic and clinical features of migraine, including prevalence of acute allodynia, were consistent with those of large scale population studies performed in several countries. In addition, the good test-retest reproducibility of TPC using TP cutoff threshold set at ≥ moderate pain response and substantial intra-rater agreement in WPH diagnosis were confirmed. Therefore, it seems that the results of this study were generalizable. As this should be considered a preliminary study, future research is warranted to evaluate whether the results could be replicated by other raters in cohorts of other nationalities and ethnicities. More refined randomized controlled trials, focusing on methodological issues relating to the determination of WPH, are warranted to elucidate the true relationship between interictal WPH and susceptibility to higher frequency of migraine attacks and to migraine onset at a younger age.

### Directions for future research

Future research should focus on identifying the pathophysiological mechanisms linking migraine and interictal WPH. Given the results of this study, patients were divided into four subtypes: 1) migraine with both acute CA and interictal WPH; 2) migraine with acute CA only; 3) migraine with interictal WPH only; and 4) migraine with neither acute CA nor interictal WPH. Direct comparison of high resolution functional MRI results between the four groups in a large-sized cohort, may delineate functional and structural alterations and characteristics of the brain related to interictal WPH and acute CA, which would lead to elucidation of the pathophysiology of migraine. As WPH is not related to the duration of disease, it is not due to barrage of headaches but is presumed to be related to the structural changes in the “migraine brain” [78]. Direct comparison using MRI between the subgroups with and without WHP may also reveal genetically determined functional and structural alterations in the “migraine brain.” New interventions can then be tested for their capacity to normalize the anatomical and functional alterations associated with migraine subtypes. However, these concepts await experimental evidence.

## Conclusion

This non-blinded cross-sectional study investigated the prevalence of interictal WPH by TPC, using positive TP with the threshold cutoff set at ≥ moderate pain response, other than QST. To our knowledge, this is the first study to show the high prevalence of interictal WPH (42%). Interictal WPH was associated with younger age at onset, female gender and higher frequency of headaches, but not duration of migraine in years, suggesting that it is a possible indicator of genetically influenced susceptibility to headache. Identification of interictal WPH in migraine in daily practice using TPC is recommended to determine tailored treatment strategies. Although the diagnostic value of TPC has declined [87], we expect that TPC, as an alternative to QST, will again be useful as a diagnostic indicator of interictal hyperalgesia to experimental pain in migraineurs.

## Additional file

Additional file 1 Original data. (XLSX 247 kb)

## Abbreviations

ACA: Acute cutaneous allodynia
CA: Cutaneous allodynia
FM: Fibromyalgia
MTPS: Manual t ender point survey
M-W: Mann-Whitney
QST: Quantitative sensory testing
TP: Tender point
TPC: Tender point count
WPH: Widespread pressure hyperalgesia
